# Significant Increase in Blood Pressure Following BNT162b2 mRNA COVID-19 Vaccination among Healthcare Workers: A Rare Event

**DOI:** 10.3390/vaccines10050745

**Published:** 2022-05-10

**Authors:** Nikolaos Syrigos, Anastasios Kollias, Dimitra Grapsa, Eleni Fyta, Konstantinos G. Kyriakoulis, Ioannis Vathiotis, Elias Kotteas, Ekaterini Syrigou

**Affiliations:** 1 Third Department of Medicine, School of Medicine, National and Kapodistrian University of Athens, Sotiria Hospital, 11527 Athens, Greece; nsyrigos@hsph.harvard.edu (N.S.); taskollias@gmail.com (A.K.); dimgrap@yahoo.gr (D.G.); elenifita1@gmail.com (E.F.); konkyriakoulis@gmail.com (K.G.K.); johnvathiotis1@gmail.com (I.V.); ilkotteas@hotmail.com (E.K.); 2 Department of Allergy, Sotiria Hospital, 11527 Athens, Greece

**Keywords:** BNT162b2 vaccine, COVID-19, blood pressure, hypertension

## Abstract

This brief report examined the frequency and characteristics of a significant blood-pressure (BP) increase after Pfizer-BioNTech BNT162b2 vaccination among healthcare workers who were advised to measure their BP at home. A total of 797 participants (mean age 48.1 ± 10.8 years, 63% women, 39% smokers) were included in the analysis. Seven participants reported an increase in their BP (three in the range of grade 2 and four in the range of grade 3 hypertension). Only one participant had a history of treated hypertension. The BP increase was observed at the end of the first week after the first dose, lasted for 3 to 4 days, and recurred promptly after the second dose. Only one case required hospitalization, mainly due to a history of cardiovascular disease (follow-up). Individuals experiencing a BP increase compared with those not reporting issues with their BP had a higher mean age and similar distribution of sex and non-smoking status. In conclusion, a significant BP increase after Pfizer-BioNTech vaccination seems to be rare and of a benign and transient nature. Monitoring the BP before and after vaccination might be advisable only for selected individuals with a high cardiovascular risk.

## 1. Introduction

The Pfizer-BioNTech BNT162b2 vaccine against COVID-19 has established a favorable safety profile and high efficacy in reducing the SARS-CoV-2 infection rates and severity of disease, based upon the findings of clinical trials and post-authorization safety monitoring [1,2,3].

One of the less well-studied side effects involves episodes of blood pressure (BP) increase, especially in the range of grade 3 hypertension (systolic/diastolic BP ≥ 180/110 mmHg; hypertensive urgencies or emergencies). Relevant data are scarce, and only three previous studies have reported that significant BP increase among vaccinated individuals is a rare event [4,5,6]. Most of the participants in these studies received the Pfizer-BioNTech vaccine. One of these studies included elderly participants [4], another healthcare workers [5], and the last one both the general population and healthcare workers [6]. Grade 3 hypertension was observed in <1% [4] to 3% [6].

This study aimed to describe the frequency and characteristics of BP increase after Pfizer-BioNTech BNT162b2 vaccination among healthcare workers.

## 2. Materials and Methods

This is an observational study evaluating BP behavior after BNT162b2 vaccination among healthcare workers of Sotiria Hospital, Athens, Greece, conducted during the second pandemic wave (December 2020–February 2021). Participants’ demographics were recorded and triplicate BP measurements were performed before receiving the first dose (use of validated automated devices with appropriate size of upper arm cuff). The average value of the last two of triplicate BP measurements was used in the analyses in line with the current European guidelines [7,8]. Participants were advised to measure their BP at home with the suggestion to use validated automated devices [9].

Age is presented as mean ± SD. Comparison between groups with respect to age was performed with a *t*-test; for the sex and smoking status distribution, a Chi-squared or Fisher’s exact test was used. All hypothesis testing was performed at a two-sided significance level of α equal to 0.05.

## 3. Results

**Figure 1:**
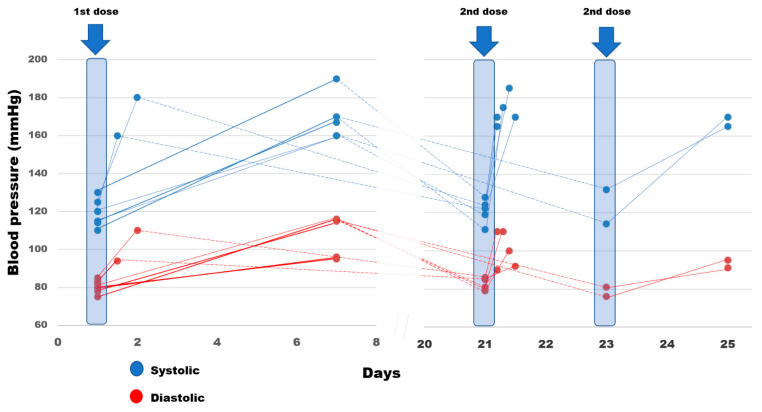
Time course of blood pressure levels in seven individuals experiencing a significant increase after vaccination. Blue highlighted columns indicate blood-pressure values measured just before the first or second dose of vaccination.

A total of 797 participants (mean age 48.1 ± 10.8 years, 63% women, 39% smokers) were included in the analysis. Three participants reported an increase in their BP in the range of grade 2 hypertension (systolic BP ≥ 160 mmHg and/or diastolic BP ≥ 100 mmHg) and four individuals in the range of grade 3 hypertension (≥180/110 mmHg). This BP increase was observed at the end of the first week after the first dose (Figure 1). Among these seven individuals, two were under treatment with antihypertensive drugs (renin-angiotensin blockers) due to a history of hypertension and myocardial infarction, respectively. The patient with a history of myocardial infarction was hospitalized mainly for close follow-up and without any acute coronary event being confirmed (hypertension urgency). Among the seven individuals experiencing a significant BP increase, three reported headache and one palpitations. The BP increase lasted for 3 to 4 days according to the individuals’ self-reports and decreased to baseline levels afterwards, which was confirmed by BP measurements before the second dose. Interestingly, all these individuals presented with a recurrence of the BP increase shortly (within a few hours) after the second dose (Figure 1). Individuals experiencing a BP increase compared with those not reporting issues with their BP (i) had a higher mean age (58.1 ± 4.1 vs. 48.0 ± 10.8 years, respectively; *p* < 0.05); (ii) similar sex distribution (3/7 (43%) vs. 288/790 (37%) females/total sample, respectively; *p* = NS), and (iii) similar distribution of non-smokers (6/7 (86%) vs. 448/790 (57%), non-smokers/total sample respectively, *p* = NS).

## 4. Discussion

The main findings of this study include the following: (i) the increase in BP levels in the context of hypertension urgency was a rare complication after Pfizer-BioNTech BNT162b2 vaccination; (ii) this side effect occurred at the end of the first week after the first dose and was followed by spontaneous resolution and prompt recurrence after the second dose; (iii) this side effect was more frequent in older individuals.

The exact prevalence of a significant increase in BP levels after mRNA-based SARS-CoV-2 vaccination is unknown. The first report is from Meylan et al. [4], who reported nine cases (median age: 73 years) of severe hypertension (grade 3) shortly (within minutes) after the administration of Pfizer-BioNTech BNT162b2 or Moderna mRNA-1273 among 12,349 patients receiving at least the first dose; eight of these patients had a history of arterial hypertension, and six were on antihypertensive medications at the time of vaccine administration [4]. Zappa et al. [5] performed an online survey among healthcare workers (n = 113; mean age 43 years) and reported that six individuals (83% treated for hypertension) presented an average rise in systolic or diastolic BP at home by ≥10 mmHg during the first five days after the first dose of Pfizer-BioNTech vaccine when compared with the five days before the vaccine. Lastly, Bouhanick et al. [6] performed a retrospective analysis of 21,909 individuals, with 37% presenting BP ≥ 140/90 mmHg 15 min after the first dose of Pfizer-BioNTech vaccine and 3.2% with grade 3 hypertension. Individuals with grade 3 hypertension were older than those with normal BP [6]. Based on the aforementioned findings, it appears that a significant increase in BP levels after Pfizer-BioNTech vaccination is a rather rare complication. Our results confirm and extend these prior findings, further suggesting that new-onset transient hypertension—and not just the worsening of pre-existing hypertension—may be observed following administration of the BNT162b2 vaccine.

One of the strengths of the current study was the description of the time course of the BP increase after vaccination by using home BP monitoring. All cases of grade 2 or 3 hypertension occurred within the first week after the first dose followed by spontaneous resolution and recurrence shortly after the second dose. The recurrence of a BP increase in the same individuals after the second dose—with shortening of the interval between the timing of vaccine administration and occurrence of the event compared to the first dose—argues in favor of a potential causal link between the vaccine and the event instead of dismissing these findings as coincidental. Notably, all the cases observed were of a transient and benign nature; hospitalization was required only in a single case (due to a history of cardiovascular disease), and all patients were fully recovered without any complications.

Moreover, one of the novelties of this study was that most individuals experiencing such BP increases were older than their counterparts. However, no significant differences were observed with respect to the sex and smoking status distribution. Interestingly, most of the individuals experiencing a significant BP increase after vaccination were non-smokers, but the number of observations was very low to allow significant differences to be indicated. Accumulating research is focused on the interaction between the SARS-CoV-2 spike protein and the nicotinic acetylcholine receptor, which could be involved in the pathology and infectivity of the SARS-CoV-2 virus [10,11], and a recent rapid systematic review showed that active smoking had a negative impact on the humoral response to COVID-19 vaccines [12].

The present study provided novel findings compared to previous studies regarding the time course of the BP increase after Pfizer-BioNTech vaccination by assessing home BP values. Moreover, it examined the relationship of the BP increase with the history of hypertension and that of smoking. On the other hand, the lack of information regarding the percentage of participants using the recommended validated automated BP monitors as well as the lack of recording home BP data according to a standardized monitoring schedule represent limitations. It should be noted that some individuals might have experienced a BP increase without documentation. Moreover, this was an observational study based on office and patients’ self-reported selected BP values in a short interval without multiple objective measurements, i.e., with ambulatory BP monitoring. Thus, some of the findings could also reflect, at least partly, the effect of the stress of vaccination (real biological or psychologically induced stress).

In conclusion, a significant BP increase after Pfizer-BioNTech vaccination seems to be rare and almost always of a benign and transient nature. Monitoring the BP before and after vaccination, especially in individuals with cardiovascular comorbidities, might be advisable. Ongoing post-marketing safety surveillance for the identification of clinically meaningful adverse reactions, including cardiovascular events, which have not been previously recognized either due to their rarity or occurrence in patient subgroups underrepresented in clinical trials, is undoubtedly essential to ensure that vaccine safety standards are rigorously met in the wider population.

## Data Availability

Data are available upon reasonable request from the corresponding author.
